# New Appliances and Things Medical

**Published:** 1911-07-29

**Authors:** 


### NEW APPLIANCES AND THINGS MEDICAL.

### STANDARD GERM FOOD.

(H. W. Murfitt and Sons, Downham Road, Ely.)

This registered preparation is stated to be made from purest wheat, and an examination bears out this statement. The granulated powder, not unlike browned biscuit dust in appearance and taste, contains a relatively large amount of mineral salts besides the usual albumins and glutens found in good wheat flour. The percentage of starch is relatively high, and the food is of course not specially suitable for infants. For children and adults it is a useful and exceedingly cheap preparation, easily prepared—though unlike most modern wheat-foods, it needs thorough boiling—and fairly easily digested.

### A VACCINATION PAD.

(The Columbia Proprietary Company, 5 Abbotts Road, Southall, W.)

The sample of Pulvinecto which has been sent to us consists of a dusting powder and a pad with attachment for use after vaccination. The powder is a mixture of boric acid, zinc oxide, and starch; the pad is a simple gauze-covered piece of soft wood. As an application to the vaccination wound it is harmless and soothing; its simplicity and cheapness are additional points in its favour. On the cover of each pad is printed a simple and lucid statement setting forth the advantages of vaccination and revaccination. Practitioners will find Pulvinecto a useful help in vaccination work.

### CULN BRASS.

(Gawthorp and Sons, 16 Long Acre, London, W.C.)

This is a special brass, stamped in a corner with the word "Culn," which is the registered mark of the firm, and admirably adapted for institutional memorial tablets. The recently unveiled tablet in the Hackney Town Hall is the work of Messrs. Gawthorp and Sons, and is a handsome and artistic example of the beautiful effects that may be obtained with this brass. The registered Albascript tablets also merit the hospital worker's attention. The lettering on these tablets is imperishable, although it is uncovered by any protecting varnish; it is a part of the tablet itself, cut in relief, the groundwork of the tablet being removed and the interstices filled up with a durable composition. Samples will be sent free on application.

### HEPTOS.

(Thomas Christy & Co., 4 Old Swan Lane, Upper Thames Street, E.C.)

Manufactured by the well-known firm the H. K. Mulford Company, of Philadelphia, "Heptos" is put up in a form that will appeal to everyone who has the necessity to prescribe or take a medicine which claims to be a laxative. "Heptos" is an effervescent, granular powder, the base of which is sodium phosphate, with which are combined lithium citrate and phenolphthalein (1 to 2 grains in each teaspoonful). The efficacy of phenolphthalein as a laxative is too well known to need comment here; prescribed in this form it is unlikely to give rise to the violent griping pain which some patients complain of when using it. "Heptos" is particularly suitable to those who desire a mild, efficacious, and reliable laxative and cholagogue.

### HAZOL.

(Allen and Hanburys, Ltd., 37 Lombard Street, E.C.)

Hazol is a spirituous antiseptic solution in which are combined the active principle of hamamelis, thymol, menthol, and eucalyptol, with the benzoates, bicarbonates, and biborates of sodium. It is in appearance a transparent ruby-red liquid, freely mixable with water in all proportions, and is a most excellent preparation for the mouth and throat. Used as a gargle or tooth-wash, it is agreeable and effective; its antiseptic properties are marked, and its use internally (in half or dram doses) is worth a trial in cases of gastric and intestinal trouble in which hamamelis or the thymol group is indicated. It may also be used as a dressing for wounds. The price is 3s. 6d. per 16-ounce bottle and 2s. for the smaller 8-oz. size.

